# Pan-Immune-Inflammation Value as a Novel Predictor of Contrast-Associated Acute Kidney Injury in Patients Treated with Primary PCI for STEMI

**DOI:** 10.3390/jcm15062456

**Published:** 2026-03-23

**Authors:** Gökhan Çiçek, Sadık Kadri Açıkgöz, Eser Açıkgöz, Servet Altay

**Affiliations:** 1 Department of Cardiology, Ankara Bilkent City Hospital, University of Health Sciences, 06800 Ankara, Türkiye; 2 Department of Cardiology, Abdurrahman Yurtaslan Oncology Education and Research Hospital, 06800 Ankara, Türkiye; 3 Department of Cardiology, Faculty of Medicine, Trakya University, 22030 Edirne, Türkiye

**Keywords:** contrast-associated acute kidney injury, ST-segment elevation myocardial infarction (STEMI), pan-immune-inflammation value, primary percutaneous coronary intervention, cardiovascular disease

## Abstract

**Background/Objectives:** Contrast-associated acute kidney injury (CA-AKI) remains an important cause of morbidity and mortality in patients undergoing procedures that require intravascular contrast administration. Therefore, the early identification of high-risk individuals is paramount, above all for ST-segment elevation myocardial infarction (STEMI) patients in need of urgent percutaneous coronary intervention (PCI). **Methods:** This retrospective study evaluated the prognostic value of the Pan-Immune-Inflammation Value (PIV), a composite inflammatory index, in predicting CA-AKI among patients presenting with STEMI who received urgent PCI within a 12 h window from the onset of symptoms. **Results:** This study recruited 2325 patient. CA-AKI was defined as a >25% or ≥0.5 mg/dL increase in serum creatinine within 48–72 h after the procedure. Patients were categorized into CA-AKI (+) and CA-AKI (−) groups. PIV levels were significantly higher in patients who developed CA-AKI (502.5 ± 324.5 vs. 264.7 ± 165.8; *p* < 0.001). ROC analysis identified a PIV cutoff value of >320, yielding an AUC of 0.753 (95% CI: 0.740–0.787; *p* < 0.001), with 67% sensitivity and 66.9% specificity. Multivariate logistic regression confirmed that PIV > 320 independently predicted CA-AKI (OR 2.118; 95% CI: 1.329–3.790; *p* < 0.001). In multivariable analysis, age, Killip class, contrast volume, and PIV > 320 were identified as independent predictors of CA-AKI. **Conclusions**: Elevated admission PIV serves as an independent and practical biomarker for predicting CA-AKI in STEMI patients undergoing PCI.

## 1. Introduction

ST-segment elevation myocardial infarction (STEMI) continues to represent one of the leading causes of mortality globally [1]. Percutaneous coronary intervention (PCI) is the primary reperfusion strategy for patients with STEMI; nevertheless, contrast-associated acute kidney injury (CA-AKI) develops in about 15–35% of cases following the procedure [2]. CA-AKI, a major cause of hospital-acquired kidney injury, is linked to prolonged renal impairment and an increased risk of mortality. The development of CA-AKI is closely associated with several recognized risk factors, such as baseline renal dysfunction, older age, and comorbidities including diabetes, hypertension, and heart failure [3]. The pathogenesis of CA-AKI is not yet fully understood; however, several studies have demonstrated a strong association between systemic inflammatory biomarkers and the development of CA-AKI, with elevated inflammatory marker levels identifying patients at higher risk of acute renal insufficiency following contrast media exposure in the setting of STEMI [4,5]. Early identification of high-risk patients is crucial for optimizing outcomes in those with STEMI undergoing PCI; therefore, reliable indicators for predicting contrast-associated acute kidney injury (e.g., using new biomarkers) in patients with acute myocardial infarction are urgently needed [6,7,8].

In recent years, it has been frequently studied that inflammation plays an important role in cardiovascular and systemic diseases. Indices that indicate inflammation are easy and accessible methods for assessing prognosis [9,10,11,12].

The Pan-Immune-Inflammation Value (PIV) combines several inflammatory markers to provide a comprehensive evaluation of the immune-inflammatory response, offering a more integrated reflection of systemic inflammation than individual parameters alone [13,14,15]. PIV has emerged as a novel marker of systemic inflammation in recent studies [16,17,18]. PIV has been found to correlate with coronary artery severity in non-ST-segment elevation myocardial infarction (NSTEMI) patients and has also been predictive of CA-AKI in this population [17,18]. While PIV has been shown to be a reliable predictor of mortality in STEMI patients [16], its association with CA-AKI in this group has not yet been established.

The objective of the present study was to examine the relationship between PIV and the occurrence of CA-AKI in STEMI patients undergoing primary PCI.

## 2. Methods

This retrospective study included 2495 consecutive STEMI patients who presented to our Emergency Department and underwent primary percutaneous coronary intervention (p-PCI) in our catheterization laboratory. The study protocol was conducted in accordance with the Declaration of Helsinki and received formal approval from the Institutional Review Board of the University of Health Sciences, Ankara Bilkent City Hospital (Reference: TABED 2-26-2053; 18 February 2026). As the study was conducted retrospectively using previously documented medical records, the need for informed consent was waived.

Patients were eligible for inclusion if they met the following criteria: (i) presentation within 12 h of symptom onset (defined as typical chest pain lasting >30 min); (ii) ST-segment elevation of ≥2 mm in at least two contiguous leads or new-onset complete left bundle branch block (LBBB); and (iii) management with primary PCI (including angioplasty and/or stent implantation).

Patients with cardiogenic shock, active infection, autoimmune diseases, hematological proliferative diseases, severe valvular heart disease, or neoplasia were excluded. A total of 170 patients were excluded because of the absence of any indication for PCI (*n* = 72), coronary bypass surgery (*n* = 69), dialysis history (*n* = 8), or missing or unavailable laboratory values (*n* = 21). Patients diagnosed with myocardial infarction with non-obstructive coronary arteries (MINOCA) or Takotsubo syndrome were excluded from the study population [19,20,21]. Thus, the final study population consisted of 2325 patients. Patients were categorized into two groups according to the occurrence of CA-AKI.

Patient demographics and cardiovascular histories—including risk factors such as smoking, hypertension, and diabetes mellitus (DM)—were retrieved from electronic medical records. Angina-to-reperfusion times were documented throughout the hospitalization period. Upon admission and prior to catheterization, an electrocardiography (ECG) was performed, and venous blood samples were collected from the antecubital vein for the measurement of complete blood counts and other serum parameters, including potassium, creatinine, and glucose.

Biochemical analyses were performed using the Roche Diagnostics Cobas 8000 c502 analyzer (Indianapolis, IN, USA). Complete blood counts were measured with a Coulter Counter LH Series (Beckman Coulter Inc., Hialeah, FL, USA). Serum creatinine levels were monitored for 72 h following PCI to assess the development of CA-AKI.

Inflammatory indices, including PIV, SII, NLR, and PLR, were calculated based on admission peripheral blood counts using the formulas detailed below. All laboratory data reflected the patients’ status at the time of hospital presentation [22]:

PIV = neutrophil count (10^9^/L) × platelet count (10^9^/L) × monocyte count (10^9^/L)/lymphocyte count (10^9^/L).

SII = neutrophil count (10^9^/L) × platelet count (10^9^/L)/lymphocyte count (10^9^/L).

NLR = neutrophil count (10^9^/L)/lymphocyte count (10^9^/L).

PLR = platelet count (10^9^/L)/lymphocyte count (10^9^/L).

All primary PCI procedures were performed following administration of aspirin and a P2Y12 inhibitor, selected at the operator’s discretion. Coronary angiography was conducted via the percutaneous radial approach. During the initial assessment of coronary anatomy, a bolus of 100 U/kg heparin was administered. Iohexol-containing contrast agent (Omnipaque, GE Healthcare, Cork, Ireland) was used in all procedures.

Diabetes mellitus (DM) was defined by a fasting blood glucose level >126 mg/dL or the current use of insulin or oral antidiabetic agents. Hypertension was established as blood pressure >140/90 mmHg or the administration of antihypertensive therapy, while hyperlipidemia was defined by a total cholesterol level ≥200 mg/dL. The estimated glomerular filtration rate (eGFR) was calculated at admission using the Modification of Diet in Renal Disease (MDRD) equation [23]; however, formal chronic kidney disease staging was omitted due to insufficient data on pre-admission renal function.

Contrast-associated acute kidney injury was defined, according to established criteria [24], as a >25% increase or an absolute rise of ≥0.5 mg/dL in serum creatinine from baseline within 48–72 h post-procedure. Additionally, left ventricular ejection fraction (LVEF) was evaluated via the modified Simpson method using a Philips Affiniti 50 echocardiography system (2–4 MHz transducer; Philips Healthcare, Andover, MA, USA).

### Statistical Analysis

Statistical analyses were conducted using SPSS version 18.0 (SPSS Inc., Chicago, IL, USA). Continuous variables are reported as mean ± standard deviation (SD) for normally distributed data or as median and interquartile range (IQR) for non-normally distributed data. Group differences were evaluated using the unpaired *t*-test or Mann–Whitney U-test, as appropriate. Categorical variables are presented as frequencies and percentages, with comparisons performed via the chi-square (χ^2^) test.

The association between PIV levels and the occurrence of CA-AKI was evaluated using logistic regression analysis, with odds ratios (ORs) and 95% confidence intervals (CIs) reported. The correlation between PIV and CK-MB was assessed using the Pearson correlation coefficient. Receiver operating characteristic (ROC) curve analysis was performed to determine the optimal PIV cut-off value for predicting CA-AKI. Multivariable logistic regression analysis was conducted to identify independent predictors of CA-AKI, including all variables with a univariate *p* value < 0.10 (age, Killip class > 1, PIV > 320, and contrast volume per 10 mL) in the model. A *p*-value < 0.05 was considered statistically significant.

## 3. Results

A total of 2325 patients were included in the present study. CA-AKI occurred in 512 patients (22.0%). Patients who developed CA-AKI were significantly older and had higher mean contrast media volume, as well as higher proportions of female gender, diabetes mellitus, hypertension, and Killip class > 1 at admission (Table 1).

Among laboratory findings, platelet counts and CK-MB were significantly higher in the CA-AKI group. Furthermore, PIV was significantly higher in patients who developed CA-AKI (502.5 ± 324.5 vs. 264.7 ± 165.8, *p* < 0.001). SII, NLR and PLR were also significantly higher in the CA-AKI group (Table 2).

**Figure 1:**
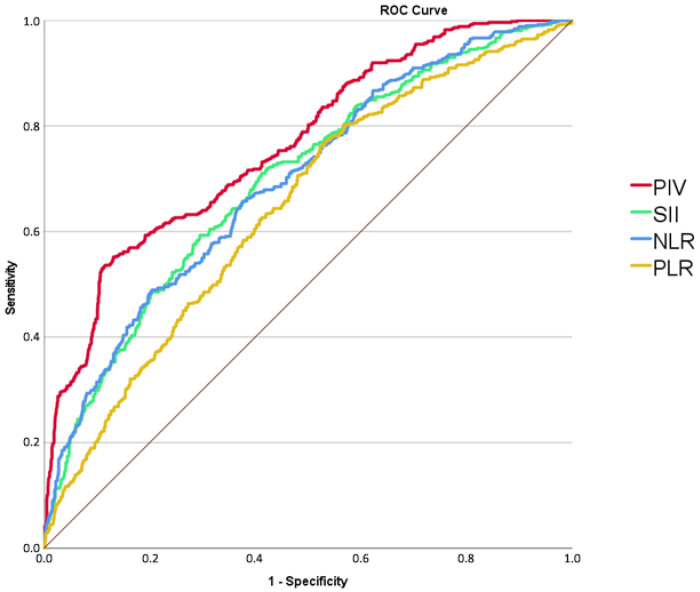
ROC curve analysis demonstrating the cut-off values of pan-immune-inflammation value (PIV), systemic immune-inflammation index (SII), neutrophil-to-lymphocyte ratio (NLR) and platelet-to-lymphocyte ratio (PLR) for the prediction of contrast-associated acute kidney injury.

In the study population, the optimal cut-off value for PIV to predict CA-AKI was >320, as determined by ROC curve analysis. This threshold yielded an area under the curve (AUC) of 0.753 (95% CI: 0.740–0.787; *p* < 0.001), with a sensitivity of 67% and a specificity of 66.9%. The receiver-operating characteristic curve analysis also demonstrated that the area under the curve was 0.697 (95% CI, 0.672–0.723; *p* < 0.001) for SII, 0.695 (95% CI, 0.670–0.721; *p* < 0.001) for NLR, and 0.645 (95% CI, 0.619–0.672; *p* < 0.001) for PLR. (Figure 1).

In multivariate logistic regression analysis, a pan-immune inflammatory value >320 was found as an independent predictor of CA-AKI (OR 2.118, 95% CI 1.329–3.790, *p* < 0.001). Age, admission Killip Class > 1 and contrast volume were other independent predictors of CA-AKI. (Table 3).

A statistically significant positive correlation was observed between CK-MB and PIV (r = 0.210, *p* < 0.001), indicating a weak association between the two parameters, as determined by the Pearson correlation coefficient.

## 4. Discussion

In this study, we demonstrated that admission PIV is independently associated with the development of CA-AKI in patients undergoing primary PCI for STEMI. Our findings suggest that PIV may serve as a practical marker for early risk stratification in this high-risk population. To our knowledge, this is the first study to specifically evaluate the association between PIV and CA-AKI in STEMI patients treated with PCI. In addition to PIV, advanced age, admission Killip class > 1, and contrast volume were also independently associated with CA-AKI.

Contrast-associated acute kidney injury remains a prevalent complication following coronary angiography and PCI. It is closely linked to prolonged hospital stays, elevated healthcare expenditures, and adverse cardiovascular and renal outcomes [25]. Despite its clinical relevance, effective preventive strategies remain limited, underscoring the importance of early identification of patients at increased risk [26]. Although several well-established risk factors for CA-AKI have been described [27,28,29], prediction in routine clinical practice remains challenging due to interindividual variability in susceptibility to renal injury after contrast exposure [30]. Fındık et al. demonstrated that composite immunonutritional and inflammatory indices may provide incremental value for risk stratification of CA-AKI in patients undergoing computed tomography in the emergency rooms [31]; in this context, our findings add clinically relevant evidence by highlighting a simple and readily available predictor of CA-AKI at emergency department presentation in patients with STEMI undergoing primary PCI. The risk of CA-AKI is particularly pronounced in STEMI patients, largely owing to greater procedural complexity, higher contrast volumes, and frequent hemodynamic instability [32]. Accordingly, current European Society of Cardiology guidelines emphasize careful risk assessment to prevent contrast-associated renal injury [33]. Risk prediction models such as the Mehran risk score are widely used to estimate the risk of contrast-associated acute kidney injury following percutaneous coronary intervention. These models incorporate several clinical and procedural variables, including hypotension, intra-aortic balloon pump use, baseline renal function, diabetes, and contrast volume [24]. Similarly, several parameters included in the Mehran risk score, or those with comparable characteristics, such as age, Killip class, and contrast volume, were also identified as independent predictors of CA-AKI in our cohort. Notably, PIV > 320 emerged as an additional independent predictor, suggesting that incorporating inflammatory status alongside traditional clinical and procedural risk factors may enhance risk stratification for CA-AKI in STEMI patients undergoing PCI. These findings align with prior evidence highlighting the relevance of systemic inflammation in the pathogenesis of contrast-associated renal injury and support the potential incremental value of composite inflammatory indices such as PIV. In this context, inflammatory biomarkers such as PIV may provide complementary information to traditional risk scores.

Consistent with prior studies, contrast volume remained an important independent predictor of CA-AKI in our analysis, highlighting the need for careful contrast management, especially in high-risk patients [34]. Similarly, Killip class ≥ 2 and advanced age, both well-established predictors of CA-AKI, were also independently associated with renal injury in our cohort [3,35]. Given the complex hemodynamic status of STEMI patients, we used the term CA-AKI to reflect the difficulty of separating contrast-related toxicity from concurrent hemodynamic and systemic factors. To reduce the influence of major competing causes of acute kidney injury, patients presenting with cardiogenic shock were excluded from the study population, thereby improving internal validity and better isolating contrast-associated renal injury. Importantly, baseline serum creatinine levels and estimated glomerular filtration rate at admission were similar between patients who developed CA-AKI and those who did not and were included in the multivariable model, suggesting that the observed association with PIV was not solely driven by differences in baseline renal function. These findings reinforce the combined contribution of hemodynamic status and baseline patient characteristics to CA-AKI development in the setting of primary PCI.

Although the precise mechanisms underlying CA-AKI are not fully elucidated, inflammation and oxidative stress play central roles in its pathophysiology. Inflammatory biomarkers, including neutrophils, monocytes, lymphocytes, and platelets, have been shown to predict adverse cardiovascular outcomes in STEMI [25,26,27,28,29,30,31,32,33,34,35,36,37,38,39,40]. Composite inflammatory indices such as PIV, SII, and NLR have therefore attracted increasing interest, including their potential association with CA-AKI [4,18,41]. PIV, which integrates neutrophil, monocyte, platelet, and lymphocyte counts into a single index [22], has demonstrated prognostic value in several malignancies while evidence in cardiovascular disease remains limited [22,42,43,44]. Inflammatory indices derived from routine blood parameters have increasingly been investigated as predictors of adverse outcomes in cardiovascular diseases [25,38,45]. Prior research has established that inflammatory markers, including the neutrophil-to-lymphocyte ratio (NLR), platelet-to-lymphocyte ratio (PLR), and systemic immune-inflammation index (SII), are significantly associated with the incidence of CA-AKI following percutaneous coronary intervention [4,18,41]. While these markers provide valuable insights into systemic inflammation, the pan-immune-inflammation value (PIV) offers a more integrative approach by simultaneously incorporating platelet, neutrophil, monocyte, and lymphocyte counts into a single parameter. This composite nature may better reflect the balance between pro-inflammatory and immune-regulatory mechanisms that contribute to renal injury following contrast exposure. Consistent with this rationale, receiver-operating characteristic (ROC) curve analysis in our study showed that the area under the curve (AUC) for PIV was higher than those of NLR, PLR, and SII, suggesting that PIV may serve as a more powerful marker for predicting CA-AKI in this patient population. Moreover, we observed a significant positive correlation between CK-MB, a marker of myocardial necrosis, and PIV. This finding highlights the pathophysiological interplay between myocardial injury, systemic inflammation, and platelet activation in the development of CA-AKI, where neutrophil-mediated oxidative stress, platelet-driven microvascular dysfunction, and impaired immune regulation collectively contribute to renal tubular injury and microcirculatory disturbances following contrast exposure.

Previous studies have shown that PIV is superior to traditional inflammatory indices in predicting cardiovascular mortality and no-reflow in STEMI patients undergoing PCI [15,16], and is associated with coronary artery disease severity in non-STEMI populations [13]. Regarding renal outcomes, Cetinkaya et al. reported an independent association between PIV and CA-AKI in patients with NSTEMI [17]; however, data in STEMI patients have been scarce. Given the heightened inflammatory response, greater myocardial injury, and more pronounced neutrophil-to-lymphocyte imbalance observed in STEMI compared with NSTEMI [31,41], the association between elevated PIV and CA-AKI in our cohort is biologically plausible. Acute systemic inflammation in STEMI may impair renal microcirculation through oxidative stress, endothelial dysfunction, and microvascular hypoperfusion, thereby increasing susceptibility to contrast-associated renal injury [45,46]. The independent association observed in our study supports the central role of inflammation in CA-AKI pathophysiology.

Importantly, PIV is derived from routine, inexpensive blood tests readily available at admission. As such, it may represent a practical adjunctive marker for early identification of STEMI patients at increased risk for CA-AKI, potentially facilitating closer monitoring, optimization of contrast use, and implementation of preventive strategies.

The findings of the present study should be interpreted in light of several limitations. This was a single-center, retrospective study, limiting generalizability and precluding causal inference. PIV was assessed only at admission, and renal function was evaluated within 1–3 days after contrast exposure; thus, delayed creatinine elevations beyond 72 h may have been missed. Furthermore, the retrospective nature of our study precluded a comprehensive evaluation of certain risk factors for CA-AKI, such as pre-existing proteinuria and exposure to nephrotoxic agents. Formal chronic kidney disease staging could not be performed due to the retrospective design and incomplete data on prior renal function, which may have resulted in residual confounding. Additionally, exclusion of patients with cardiogenic shock may limit the applicability of our findings to the sickest STEMI populations. Post-discharge renal and clinical outcomes were unavailable. An independent external validation cohort was not available; therefore, external validation is required before clinical implementation. Another limitation of our study is the absence of the Mehran risk score, as some essential variables required for its calculation were not available due to the retrospective design. These limitations highlight the need for larger, multicenter, prospective studies.

## 5. Conclusions

This study demonstrated for the first time that elevated PIV at admission is independently associated with the development of CA-AKI in STEMI patients undergoing PCI. Prospective, large-scale studies are warranted to validate these findings and to further elucidate the clinical utility of PIV in risk stratification for STEMI patients.

## Figures and Tables

**Table 1:** Baseline characteristics of study patients.

Variables	CA-AKI (−) (*n* = 1813)	CA-AKI (+) (*n* = 512)	*p* Value
Age (years)	54.87 ± 12.04	58.03 ± 14.51	0.033
Male gender, *n* (%)	1539 (84.9)	410 (80.1)	0.009
Diabetes mellitus, *n* (%)	403 (22.1)	135 (26.6)	0.046
Hypertension, *n* (%)	677 (39.2)	222 (45.4)	0.014
Smoking, *n* (%)	1066 (63.1)	279 (60.1)	0.240
Previous PCI, *n* (%)	137 (7.6)	39 (7.6)	0.985
Killip class > 1, *n* (%)	49 (4.1)	34 (11.7)	<0.001
LVEF (%)	48.65 ± 10.74	47.16 ± 11.83	0.166
Contrast volume (mL)	232.69 ± 87.18	241.89 ± 91.42	0.019
Culprit lesion, *n* (%)			
LMCA	8 (0.4)	2 (0.3)	0.355
LAD	763 (42.0)	241 (47.0)	
CX	304 (16.8)	87 (16.9)	
RCA	896 (49.4)	24 (4.68)	

Abbreviations: CA-AKI, Contrast-associated acute kidney injury; add PCI; percutaneous coronary intervention; LVEF, left ventricular ejection fraction; LMCA, left main coronary artery; LAD, left anterior descending artery; CX, circumflex artery; RCA, right coronary artery.

**Table 2:** Laboratory findings of the patients.

Variables	CA-AKI (−) (*n* = 1813)	CA-AKI (+) (*n* = 512)	*p* Value
Admission creatinine (mg/dL)	0.915 ± 0.253	0.989 ± 0.218	0.104
Peak creatinine (mg/dL)	1.094 ± 0.218	1.618 ± 0.754	<0.001
Total cholesterol (mg/dL)	181.68 ± 39.89	187.38 ± 46.54	0.097
LDL-C (mg/dL)	111.42 ± 25.28	114.86 ± 31.87	0.102
HDL-C (mg/dL)	39.83 ± 8.36	40.79 ± 12.12	0.616
Triglyceride (mg/dL)	161.07 ± 81.03	158.31 ± 74.96	0.571
Glucose (mg/dL)	129.26 ± 50.17	133.68 ± 74.31	0.766
Hemoglobin (g/dL)	13.57 ± 1.70	13.39 ± 1.97	0.435
GFR (mL/min)	94.5 ± 12.5	89.0 ± 14.0	0.123
WBC (×10^3^/µL)	12.35 ± 3.35	12.70 ± 3.32	0.424
Platelets (×10^3^/µL)	247.51 ± 61.25	267.86 ± 77.65	0.004
Neutrophil (×10^3^/µL)	8.93 ± 3.44	9.64 ± 3.18	0.101
Lymphocyte (×10^3^/µL)	2.00 ± 0.81	1.80 ± 0.81	0.068
Monocyte (×10^3^/µL)	0.76 ± 0.35	0.67 ± 0.36	0.073
PIV	264.7 ± 165.8	502.5 ± 324.5	<0.001
SII	1498.5 ± 1092.2	2431.1 ± 1785.7	<0.001
NLR	5.85 ± 3.79	9.25 ± 6.06	<0.001
PLR	158.9 ± 80.7	203.3 ± 107.2	<0.001
Peak CK-MB (IU/L)	208.9 ± 157.1	235.5 ± 188.3	0.004
K (mmol/L)	4.10 ± 0.49	4.01 ± 0.61	0.346

Abbreviations: HDL-C, high-density lipoprotein cholesterol; LDL-C, low-density lipoprotein cholesterol; GFR, Glomerular filtration rate; WBC, white blood cell; PIV, pan-immune-inflammation value; CA-AKI, Contrast-associated acute kidney injury; NLR, neutrophil-to-lymphocyte ratio; PLR, platelet-to-lymphocyte ratio; SII, systemic immune-inflammation index; CK-MB, creatinine kinase-MB; K; potassium.

**Table 3:** Independent Predictors of CA-AKI in logistic regression analysis.

Factor	Univariable OR (95% CI)	*p* Value	Multivariable OR (95% CI)	*p* Value
Age	1.046 (1.009–1.073)	0.004	1.025 (1.012–1.039)	0.001
Male gender	0.912 (0.569–1.461)	0.702	-	-
Diabetes	1.401 (0.942–2.083)	0.096	1.218 (0.777–1.912)	0.368
Hypertension	1.201 (0.838–1.720)	0.319	-	-
Admission creatinine	0.768 (0.454–1.299)	0.325	-	-
Total cholesterol	1.004 (0.996–1.012)	0.370	-	-
LDL-C	1.003 (0.993–1.013)	0.556	-	-
Glucose	1.002 (0.998–1.004)	0.059	-	-
Killip class > 1	3.457 (2.476–4.828)	<0.001	1.786 (1.038–3.122)	0.034
PIV > 320	2.296 (1.451–3.694)	<0.001	2.096 (1.286–3.812)	<0.001
Contrast volume (per 10 mL)	1.178 (1.041–1.311)	<0.001	1.085 (1.025–1.147)	0.028
Peak CK-MB	1.004 (0.997–1.009)	0.08	-	-

Abbreviations: CA-AKI, Contrast-associated acute kidney injury; LDL-C, low-density lipoprotein cholesterol; PIV, pan-immune-inflammation value; CK-MB, creatinine kinase-MB; OR, Odds Ratio; CI, Confidence Interval.

## Data Availability

The datasets generated and analyzed during the current study are available from the corresponding author upon reasonable request. G.Ç. had full access to all the study data and assumes responsibility for the integrity and accuracy of the data analysis.
